# Enviromics crosstalk between internal and external plant environments for enhanced adaptation and *de novo* domestication

**DOI:** 10.1002/imt2.70143

**Published:** 2026-06-09

**Authors:** Lin‐An Zhang, Xuan Dong, Kai Liu, Nicolas Gonzalez, Jose Manuel Estevez, Wen‐Xue Li, Xing Wang Deng, Feng Yu, Yunbi Xu

**Affiliations:** ^1^ Longping Agricultural College, State Key Laboratory of Chemo/Biosensing and Chemometrics, College of Biology Hunan University Changsha China; ^2^ State Key Laboratory of Wheat Improvement, Peking University Institute of Advanced Agricultural Sciences Shandong Laboratory of Advanced Agricultural Sciences in Weifang Weifang Shandong China; ^3^ State Key Laboratory of Crop Gene Resources and Breeding, Institute of Crop Sciences Chinese Academy of Agricultural Sciences Beijing China; ^4^ Yuelushan Laboratory Changsha China; ^5^ Fundación Instituto Leloir and IIBBA‐CONICET Buenos Aires Argentina; ^6^ ANID ‐ Millennium Science Initiative Program‐Millennium Institute for Integrative Biology (iBio) Santiago, Chile and Centro de Biotecnología Vegetal, Facultad de Ciencias de la Vida Universidad Andres Bello Santiago Chile; ^7^ BGI Bioverse Shenzhen China

## Abstract

Climate change demands accelerated plant adaptation and *de novo* domestication. Yet current enviromics focuses disproportionately on external environments, neglecting internal dynamics—gene expression, metabolic flux, and signal transduction—within predictive envirotyping frameworks. This gap constrains plant‐environment adaptation research and crop improvement. Integrating multi‐scale envirotyping with plant‐environment interaction networks could catalyze a paradigm shift from empirical selection to mechanism‐informed design breeding. Four challenges remain: (1) constructing adaptive multi‐dimensional networks, (2) engineering transgenerational epigenetic reprogramming, (3) scaling domestication pipelines, and (4) predicting adaptive trajectories. Future efforts should converge on five domains: high‐throughput microprobe envirotyping arrays, spatiotemporally resolved multi‐omics, decoding epigenetic memory carriers, artificial intelligence (AI)‐guided genome design, and phenotype prediction models. Ultimately, advancing from multi‐omics dissection and mechanistic interpretation to targeted *de novo* design will enable the precise engineering of crop adaptive responses to environmental change.

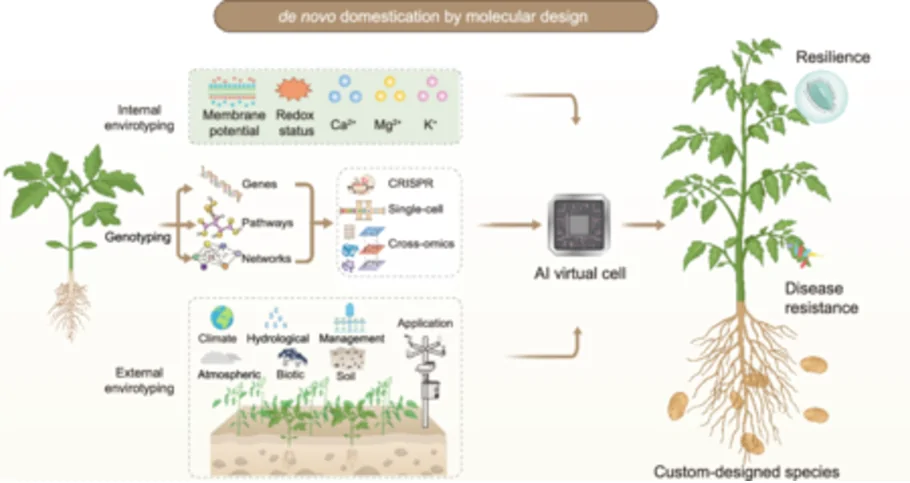

Plant phenotypes arise from genotype × environment interaction (GEI), yet the environment has been treated merely as a background factor rather than a dimension amenable to systematic envirotyping and predictive manipulation [1, 2]. Global climate change demands adaptive mechanisms translated into breeding and *de novo* domestication to enhance crop climate resilience. However, genomic‐assisted breeding is constrained by uncharacterized environments: classical GEI models treat the environment as a black box, precluding mechanistic resolution. The absence of characterized endogenous environmental response mechanisms in wild germplasm further undermines the theoretical foundation for environmental adaptation in *de novo* domestication.

To overcome this limitation, we propose a multi‐scale environmental continuum framework partitioning the plant environment into internal and external domains. At the organism scale, the epidermis serves as the operational boundary: the external environment comprises the ambient atmosphere and soil matrix outside this boundary, while the internal environment encompasses all tissue spaces within it. Grounded in *milieu intérieur* and systems biology, physicochemical conditions at each scale are defined as environments relative to their respective reference frames, rendering the internal environment an operationally measurable domain amenable to systematic envirotyping. Plant environments thus form a cross‐scale continuum quantifiable as *E* = *E*_internal_ + *E*_external_ + (*E*_internal_ × *E*_external_), where *E*_internal_ spans intracellular to tissue‐organ scales, E_external_ represents soil‐climate‐biotic factors, and the interaction term captures cross‐scale signal coupling mediated through structural interfaces. This framework deciphers the environmental black box into targetable predictive parameters for crop improvement and *de novo* domestication.

## FOUNDATIONS OF ENVIROMICS

### Internal and external enviromics

**Figure 1:**
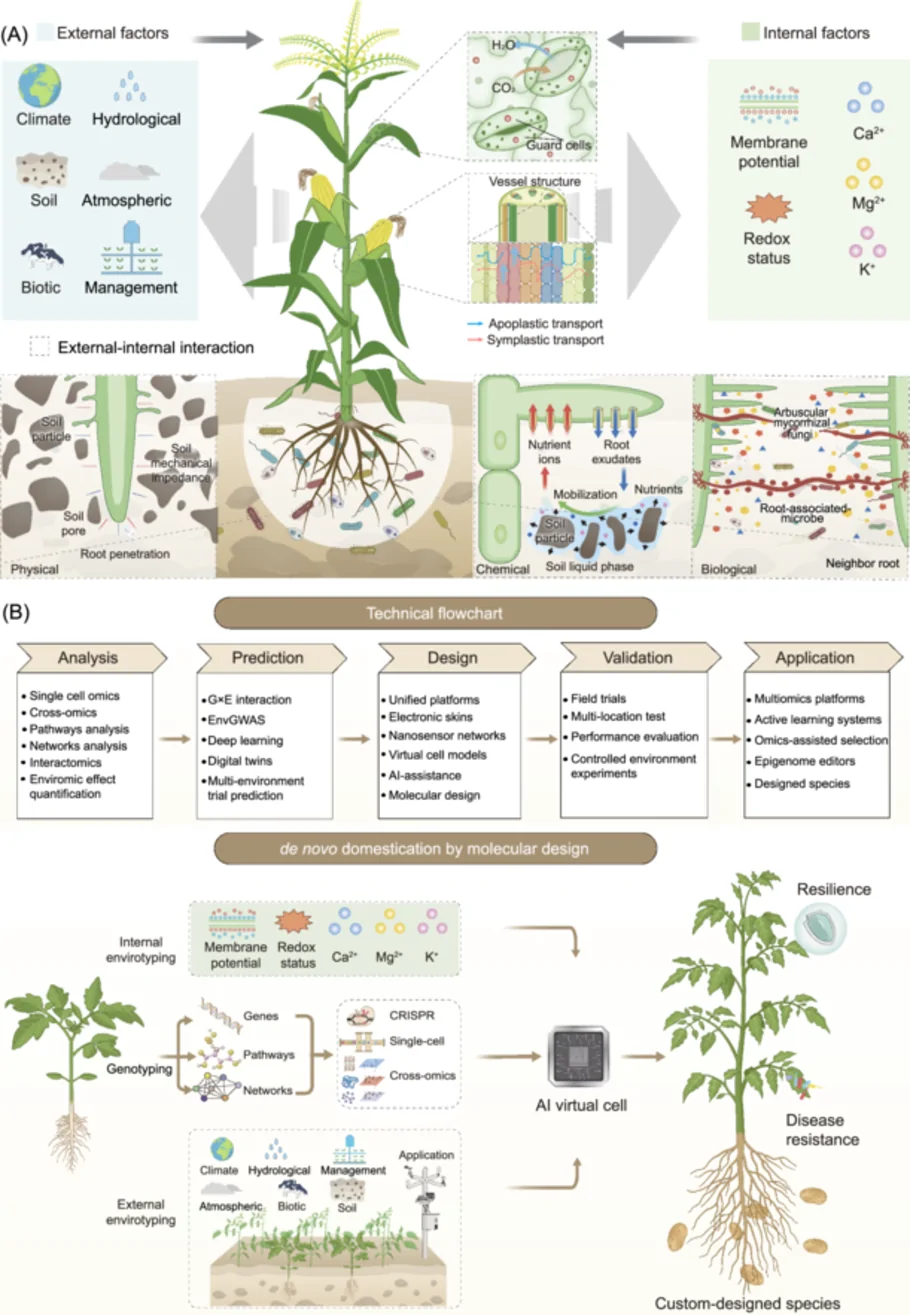
Internal‐external environmental crosstalk and *de novo* domestication design. (A) Internal‐external environmental crosstalk. The factors are categorized into external and internal factors, with their interaction mediated by organized interfacial systems. External envirotype factors include climatic, hydrological, edaphic, atmospheric, biotic, and management factors. The internal factors encompass ion concentrations, membrane potential, ROS levels, and specific factors (Table S1). Dashed boxes represent internal‐external crosstalk in the shoot (leaf) and root [3]. (B) Technical workflow for *de novo* domestication via internal‐external molecular design. The workflow proceeds sequentially through Analysis → Prediction → Design → Validation → Application. *De novo* domestication via molecular design can be implemented across internal and external envirotypes at multiple scales. In the analysis phase, internal envirotyping data (pH, ion gradients, ROS, membrane potential) and external envirotyping data (climate, soil, biotic factors) are integrated. Multi‐omics integration, AI‐guided predictive modeling, and microsensor networks are employed to forecast phenotypic outcomes and design stress‐tolerant, high‐yielding crops. Novel domesticates are engineered to integrate desirable traits from multiple progenitor species.

Enviromics is operationally defined as the integrated study of multi‐scale environmental factors governing plant growth and adaptation. It encompasses internal (*E*_internal_) and external (*E*_external_) environments, interdependent domains constituting the environmental context for GEI and phenotypic plasticity (Figure 1A,B), with the epidermis as the organism‐level boundary.

External enviromics leverages remote sensing platforms (satellite, unmanned aerial vehicle (UAV), ground‐based sensors) for large‐scale monitoring of climate, soil, and biotic factors [1, 4]. Transformer‐based architectures, such as GEFormer model GEI through attention mechanisms, improve prediction accuracy by 2.92% for specific traits and over 20% for hybrid prediction in untested environments [5], yet rely exclusively on external data (Table S1).

Internal enviromics employs electrochemical microsensors and genetically encoded biosensors to envirotype physicochemical conditions across intracellular, intercellular, and tissue‐organ domains. Microneedle electrochemical sensors enable minimally invasive continuous ion monitoring in the internal environment, though stability and field deployability remain unresolved [6] (Table S1).

Interfacial compartments such as the periarbuscular space (PAS) and peribacteroid space (PBS) exemplify microdomains with physicochemical parameters (pH, ion concentrations) that are neither cytosolic steady‐state values nor bulk environmental values [7, 8]. Rather than introducing an additional *E*_interfacial_ domain, we treat these as specialized environments within *E*_internal_, acknowledging their intermediate nature without proliferating theoretical categories. These atypical internal environments bear signatures of external input, preserving envirotyping utility while accommodating biological complexity.

Operationalizing this partitioning requires demonstrable measurability and experimental separability. *E*_internal_ and *E*_external_ are restricted to physicochemical states measurable by envirotyping. Independent measurement is achieved through controlled perturbations: (i) fixed *E*_external_ (controlled atmospheric CO_2_ with substomatal microelectrode readout), (ii) fixed G background (Near‐isogenic lines with stepwise salinity gradients, xylem Na^+^/K^+^ quantified by microneedle electrodes/inductively coupled plasma mass spectrometry).

### Intracellular, intercellular, and tissue‐organ enviromics

Internal envirotyping systematically quantifies multi‐scale dynamic physicochemical states endogenous to individual plants, operationalized across three hierarchical scales (Figure S1). This hierarchy is grounded in the scale‐relativity principle: at each scale, the physicochemical conditions surrounding the focal subsystem constitute its environment, transforming abstract conceptual layers into experimentally accessible modules (Table S1).

At the intracellular scale, the cytosol, nucleoplasm, and organellar lumina constitute the physicochemical environment for macromolecular processes. Internal envirotyping quantifies dynamic parameters regulating gene expression and metabolic flux: cytosolic pH, free ion concentrations (K^+^, Ca^2+^, Cl^−^, and NO_3_^−^ across compartments, spanning mM to μM ranges [9]), redox potentials, adenylate energy charge (ATP/ADP/AMP), and organelle‐specific states (mitochondrial membrane potential, chloroplast NADPH/NADP^+^ ratio, vacuolar pH).

At the intercellular scale, the plasma membrane serves as the intracellular‐intercellular interface, while the apoplast and plasmodesmata mediate symplastic transport and apoplastic diffusion. Internal envirotyping quantifies apoplastic pH gradients (5.0−6.5), ion gradients (K^+^, Ca^2+^, Cl^−^, and NO_3_^−^), gas partial pressures (O_2_, CO_2_, and ethylene), and signaling molecule concentrations (auxin, abscisic acid (ABA), and peptide gradients). Core modules include (Figure S2): (1) cell wall‐plasma membrane mechanosensing (*FER* receptor kinase‐*RALF* (rapid alkalinization factor), mid1‐complementing activity channels), (2) plasmodesmata gating (callose deposition, *PDLP5* (plasmodesmata‐localized protein 5) [10]), (3) calcium decoding (*CBL* (calcineurin B‐like protein)‐*CIPK* (CBL‐interacting protein kinase), *CDPK* (Ca^2+^‐dependent protein kinase) [11]), and (4) apoplastic barriers (casparian strip proteins, suberin [12]). These modules form an integrated network that remodels the internal environment within minutes.

At the tissue‐organ scale, multicellular functional units (vascular tissues, photosynthetic tissues) operate as integrated systems for long‐distance transport and gas exchange. Vascular differentiation creates distinct internal environments: xylem sap (apoplastic, tension‐driven) versus phloem sap (symplastic, pressure‐driven, and sugar‐enriched). Tissue‐layer specialization establishes hormone‐driven radial gradients, exemplified by auxin‐cytokinin antagonism across vascular cell files [13]. These gradients achieve environmental diversification through differential recruitment of common regulatory modules across cell types, repurposed from apical dominance to leaf hydathodes, floral anthers, and root apices [14].

### Internal envirotyping for predictive crop improvement

Beyond operational measurability, the analytical utility of internal envirotyping lies in transcending the background‐dependency that plagues the development of component‐level genetic markers. Traditional quantitative trait locus (QTL) and candidate gene mapping identify single loci, yet their effects depend on interactions with both external and internal environmental factors. In genetic backgrounds lacking these components, candidate gene and QTL effects fluctuate dramatically, thereby destabilizing marker‐assisted selection. In contrast, by considering *E*_internal_ parameters (such as xylem Na^+^, vacuolar Na^+^/K^+^), genetic mapping represents integrated multi‐gene network outputs via many‐to‐one genotype‐to‐phenotype mapping. For example, regardless of specific gene combinations, the maintained physicochemical state directly constrains salt tolerance [15]. Consequently, genetic mapping with *E*_internal_ factors accounted for is substantially less background‐dependent than single‐locus mapping, and thus integrating genotype with both internal and external envirotypes yields background‐robust selection criteria.

Traditional physiological measurements dominate the genotype‐phenotype binary framework, recording plant symptoms without identifying environmental drivers, thus failing to establish causal chains from internal environments to molecular networks. This perpetually confines GEI to black‐box descriptions. Envirotyping is defined as the operational process of quantifying, standardizing, and classifying physicochemical parameters into envirotype variables for predictive modeling [1]. Internal envirotyping deconstructs these parameters within intracellular, intercellular, and tissue‐organ environments into quantifiable, comparable, and standardized envirotypic variables.

These variables function as regulatory inputs for genetic programs: decomposing drought into *E*_external_ (soil water deficit severity) and *E*_internal_ (xylem O_2_, cytosolic ABA‐induced Ca^2+^ signature) identifies the specific internal envirotype response differentiating genotypes, advancing from correlation to causality.

Emerging microprobe technologies promise high‐resolution internal envirotyping. Specifically, electrochemical microsensors detect apoplastic pH, K^+^, Ca^2+^, and O_2_ gradients at cellular resolution; non‐invasive micro‐test technology (NMT) achieves non‐destructive, *in vivo* monitoring of transmembrane ion fluxes; fiber‐optic microprobes detect reactive oxygen species (ROS) and hormone distributions; and nanowire sensors enable multiplexed metabolic flux detection in vascular tissues. Complementing these physical microprobe tools, genetically encoded biosensors (e.g., green fluorescent protein calmodulin peptides, Forster resonance energy transfer sensors) enable non‐invasive imaging of cellular dynamics, while mass spectrometry imaging (matrix‐assisted laser desorption/Ionization mass spectrometry imaging, cryogenic nanoscale secondary ion mass spectrometry) and single‐cell omics resolve cell‐type‐specific metabolic responses [16]. In parallel, advanced imaging (selective plane illumination microscopy, 4Pi structured illumination microscopy) enables real‐time visualization [17].

Though largely confined to laboratory settings, next‐generation microneedle‐based electrochemical sensors are breaking this boundary: 3D‐printed solid microneedle arrays achieve near‐Nernstian pH response for continuous drought monitoring [18], demonstrating proof‐of‐concept for field‐deployable internal envirotyping.

## CROSSTALK MECHANISMS AND MULTI‐OMICS INTEGRATION

### Internal‐external enviromics communications

**Figure 2:**
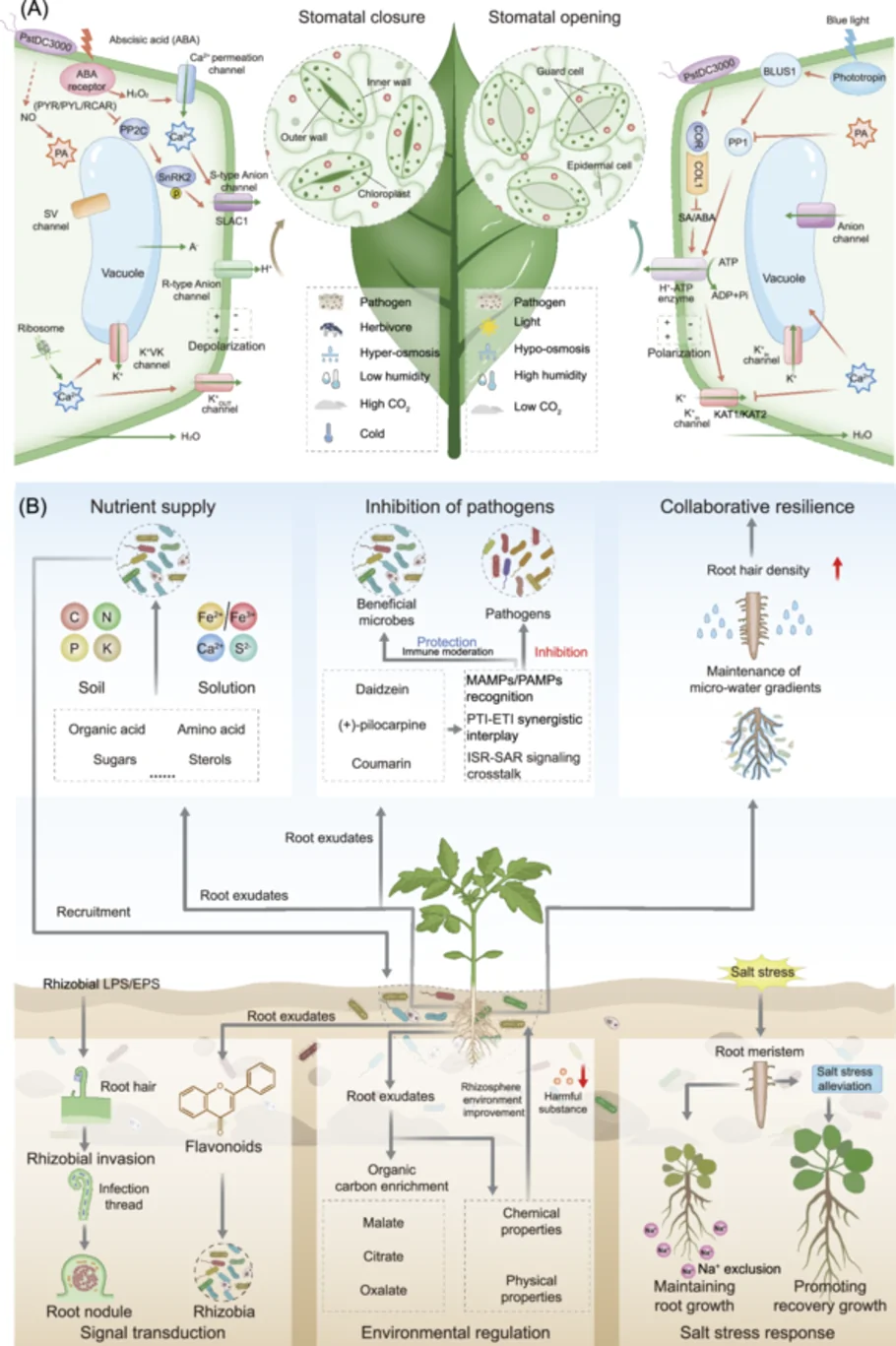
Plant stomatal movement and rhizosphere interaction regulatory network. (A) Stomatal movement regulation: The left panel illustrates the ABA‐mediated stomatal closure pathway, including ABA receptor activation, Ca^2+^ signaling, and anion channel opening; the right panel shows the mechanism of stomatal opening induced by blue light and other signals, involving H^+^‐ATPase polarization and K^+^ influx; the central panel summarizes environmental and biotic stress factors that regulate stomatal aperture. Red arrows denote signal transduction and regulatory cascades from upstream stimuli (e.g., hormones, light) to downstream effectors. Green arrows indicate substance transport and metabolic flux, including transmembrane ion/water movement and ATP hydrolysis. T‐bars (⊣) mark inhibitory interactions. The phosphorylation label *p* indicates an activated protein state. (B) Rhizosphere interaction and environmental adaptation network: This module includes root exudate‐mediated nutrient recruitment, beneficial microbial enrichment and pathogen inhibition, salt stress alleviation, and root hair density regulation, revealing that plants achieve environmental adaptability and collaborative resilience through rhizosphere environment modulation [19]. ABA, abscisic acid; ETI, effector‐triggered immunity; EPS, exopolysaccharide; ISR, induced systemic resistance; G×E, genotype × environment; LPS, lipopolysaccharide; PstDC3000, *Pseudomonas syringae* pv. tomato DC3000; EnvGWAS, environment genome‐wide association study; PA, phosphatidic acid; PTI, pattern‐triggered immunity; SAR, systemic acquired resistance.

Crosstalk in plant enviromics is not a direct exchange of environmental information, but rather cross‐scale signal conversion and material exchange mediated by interface compartments. Stomata regulate gas exchange: external factors (light, CO_2_, humidity, and temperature) trigger internal signaling (e.g., ABA‐mediated responses), while carbon status feeds back to modulate aperture. The phyllosphere enables microbial‐physiological interactions above ground. Root systems sense gradients via hormonal networks and recruit beneficial microbes through rhizosphere secretions. These interfaces collectively form the structural basis for phenomics‐enviromics crosstalk (Figure 2A,B).

This crosstalk functions across multiple scales: physiological (gas exchange, ion flux), developmental (root‐shoot allocation, stress trajectories), and evolutionary (local adaptation signatures in envirotype‐genotype covariation). Interface compartments are the physical sites of signal conversion—straddling the internal‐external boundary to permit environmental entry, enable simultaneous signal capture and intracellular response, and serve as tunable GEI loci. Quantifying crosstalk dynamics bridges descriptive biology to predictive envirotypic modeling.

This predictive capacity transforms precision agriculture from passive *E*_external_ manipulation to active engineering of multiscale environmental interaction nodes. By modulating stomatal aperture dynamics and rhizosphere secretion gradients, these interfaces function as “smart filters,” structural boundaries with genetically encoded, dynamically adjustable permeability, that actively regulate environmental exchange without altering *E*_external_, achieving precision envirotype design at cell‐tissue scales.

### Modular regulation of multi‐omics integration

The fundamental contribution of enviromics lies in transforming the environment from an unobservable background into quantifiable parameters, enabling their integration with molecular biological signal modules (perception, amplification, coordination, and output) to bridge statistical correlation to mechanistic prediction.

At the root‐soil interface, root hair growth regulated by ROP GTPases, actin dynamics, and vesicle trafficking determines water and nutrient acquisition efficiency; root border cells form an interface for defense and signal exchange. At the shoot‐atmosphere interface, stomatal movement integrates light, CO_2_, and ABA signals. The *OST1‐CPK15‐SLAC1* pathway decomposes drought adaptation into independently optimizable modules of perception, amplification, and output. High temperature acts through *phyB‐PIF4* to integrate light and temperature signals, redefining thermomorphogenesis as an active developmental program. Soil nitrate signaling via *NRT1.1* phosphorylation couples auxin transport to root system architecture, while stomatal density undergoes epigenetic remodeling in response to atmospheric CO_2_ and humidity.

Collectively, enviromics integrates dispersed molecular discoveries into modular interface networks, revealing regulatory logic inaccessible to black‐box GEI models—hierarchical signal integration and conflict resolution across cell‐tissue‐organ scales. Quantitative molecular‐environment relationships yield mechanistic, predictive understanding and quantifiable engineering targets, transforming adaptation from statistical correlation into actively designable outputs.

## *DE NOVO* DOMESTICATION AND FUTURE PERSPECTIVES

### Multi‐omics integration for *de novo* domestication

Plant‐environment interactomics deciphers molecular networks underlying environmental signal transduction, providing a mechanistic basis for engineering plant resilience. We advance the envirotyping framework to an integrated paradigm harnessing multi‐omics data and precision breeding technologies for predictive design in *de novo* domestication.

Tiered envirotyping and methodological integration involves four interconnected strategies. (1) Multi‐omics coupling identifies domestication targets: ionomics‐transcriptomics reveals *G2‐like* (G2‐like transcription factor), *bHLH* (basic helix‐loop‐helix), and *HD‐ZIP* (homeodomain‐leucine zipper) transcription factors transducing phosphorus signals into intracellular reprogramming; CRISPR (clustered regularly interspaced short palindromic repeats)‐validated loci connect interface sensing with gene regulation. (2) Sensor‐transcriptome integration enables performance prediction: field sensor networks refine external envirotyping, single‐cell transcriptomics distinguishes stress‐responsive cell populations, and genome‐environment association prioritizes editing targets. (3) Epigenome‐transcriptome coupling quantifies heritable chromatin states: DNA methylation, histone modifications, and small RNAs conferring transgenerational tolerance. Salt and cold stresses induce cytosine‐hydrogen‐hydrogen and cytosine‐guanine (CG) hypomethylation in rice, priming jasmonic acid (JA) pathway genes. Methylome‐transcriptome integration identifies stress‐responsive differentially methylated regions; targeted methylation editing validates causal epivariants [20]. (4) Microbiome association restores beneficial interactions: wild *Solanum pennellii* recruits phosphate‐solubilizing microbes via root exudates, whereas cultivated varieties lose this capacity. Engineering flavonoid biosynthesis (*OsPAL*, *OsC4H*) enhances rhizobacterial biofilm formation and biological nitrogen fixation, providing operational microbiome targets.

The five‐stage workflow (Analysis → Prediction → Design → Validation → Application) integrates envirotyping, multi‐omics, and precision editing, connecting molecular networks with field environments via AI virtual cell models. Analysis combines tiered envirotyping and multi‐omics profiling of wild germplasm. Prediction integrates envirotyping data and genomic information via machine learning. Design deploys CRISPR, methylation editing, and microbial engineering (Figure 1B). Validation combines controlled‐environment phenotyping with multi‐environment testing, using envirotyping to quantify performance variation across internal environments. Application updates envirotyping parameters and prediction models based on validation data, with active learning driving continuous optimization.

### Challenges and perspectives

Realizing plant digital twins and AI‐guided genome design requires upgrading the five‐stage workflow from discrete operations to a continuous, automated system. This depends on transitioning enviromics from static measurement to real‐time, multi‐scale sensing.

We propose a technology readiness level (TRL) framework mapping this incremental process: proof‐of‐concept for multi‐scale modeling (NeRF‐based 3D reconstruction, Crops *in silico*), component validation for standardizing envirotyping and phenotyping, system integration via physics‐informed neural networks and sensor fusion algorithms, and operational deployment targeting closed‐loop optimization. The critical bottleneck is non‐invasive internal sensing; microneedle‐based electrochemical sensors, red‐shifted biosensors, and miniaturized rhizosphere arrays provide interim solutions.

Priority directions include: (1) standardizing envirotyping protocols and global databases; (2) advancing microprobe sensor arrays; (3) elucidating cross‐scale signal integration; and (4) establishing global collaborative networks across latitudes, altitudes, and edaphic gradients. Success depends on acknowledging that scalable internal sensing remains in its infancy, and on building integrative capabilities incrementally.

The ultimate vision of enviromics is not to replace field trials, but to transform them from trial‐and‐error exploration into hypothesis‐driven precision intervention. Embedding tiered envirotyping within digital twins enables pre‐screening millions of genotype‐environment combinations in silico, deploying only optimal strategies in real fields. This is the inevitable path for *de novo* domestication to advance from empiricism to mechanistic science.

## AUTHOR CONTRIBUTIONS

**Lin‐An Zhang**: Investigation; writing—original draft; visualization; writing—review and editing. **Xuan Dong**: Writing—review and editing. **Kai Liu**: Data curation. **Nicolas Gonzalez**: Data curation. **Jose Manuel Estevez**: Data curation. **Wen‐Xue Li**: Writing—review and editing. **Xing Wang Deng**: Writing—review and editing. **Feng Yu**: Conceptualization; methodology; writing—review and editing. **Yunbi Xu**: Conceptualization; methodology; writing—review and editing; investigation; writing—original draft; visualization.

## CONFLICT OF INTEREST STATEMENT

The authors declare no conflicts of interest.

## ETHICS STATEMENT

No animals or humans were involved in this study.

## Supporting information


**Figure S1:** Hierarchical reference‐frame strategy for partitioning plant environments in the enviromics framework.
**Figure S2:** Plant cell wall sensing and intercellular transport regulatory network.

**Table S1:** Internal and external envirotyping.

## Data Availability

Data sharing not applicable to this article as no datasets were generated or analyzed during the current study. No new data or code were generated in this study. All data discussed are from previously published studies, which are cited in the reference list. Supplementary materials (figures, tables, graphical abstract, slides, videos, Chinese translated version, and updated materials) may be found in the online DOI or iMeta Science http://www.imeta.science/.
