# Supplementary material for: Enviromics crosstalk between internal and external plant environments for enhanced adaptation and de novo domestication
Source: Imeta. 2026 Jun 9;5(4):e70143. doi: 10.1002/imt2.70143 (PMC13519095; doi:10.1002/imt2.70143)
Supplement: Supplementary file 1 [file IMT2-5-e70143-s002.docx]

Supporting information to

**Enviromics crosstalk between internal and external plant environments for enhanced adaptation and *de novo* domestication**

**Running title**: Enviromics-driven internal and external environment crosstalk

Lin-An Zhang^1, 2, 3, 4#^, Xuan Dong^1#^, Kai Liu^1^, Nicolas Gonzalez^5^, Jose Manuel Estevez^5, 6^, Wen-Xue Li^3^, Xing Wang Deng^2^, Feng Yu^1, 4*^, Yunbi Xu^2, 3, 7*^

^1^Longping Agricultural College, State Key Laboratory of Chemo/Biosensing and Chemometrics, College of Biology, Hunan University, Changsha 410082, China

^2^State Key Laboratory of Wheat Improvement, Peking University Institute of Advanced Agricultural Sciences, Shandong Laboratory of Advanced Agricultural Sciences in Weifang, Shandong 261325, China

^3^State Key Laboratory of Crop Gene Resources and Breeding, Institute of Crop Sciences, Chinese Academy of Agricultural Sciences, Beijing 100081, China

^4^Yuelushan Laboratory, Changsha 410082, China

^5^Fundación Instituto Leloir and IIBBA-CONICET, Av. Patricias Argentinas 435, Buenos Aires C1405BWE, Argentina

^6^ANID - Millennium Science Initiative Program-Millennium Institute for Integrative Biology (iBio) Santiago, Chile and Centro de Biotecnología Vegetal, Facultad de Ciencias de la Vida, Universidad Andres Bello, Santiago 8320000, Chile

^7^BGI Bioverse, Shenzhen 518083, China

^#^These authors contributed equally: Lin-An Zhang, Xuan Dong

*Correspondence: yunbi.xu@pku-iaas.edu.cn, xuyunbi@caas.cn (Yunbi Xu); feng_yu@hnu.edu.cn (Feng Yu)

**Figure S1 Hierarchical reference-frame strategy for partitioning plant environments in the enviromics framework.** The nested boundary system demarcates E_internal_ and E_external_ across biological scales. (A) Plant scale: epidermis as the E_internal_/E_external_ boundary. (B) Intracellular scale: plasma membrane-enclosed cytosol and organellar lumina as the physicochemical environment for subcellular machinery. (C) Intercellular scale: plasmodesmata and apoplast as symplastic/apoplastic communication channels. (D) Tissue-organ scale: vascular and photosynthetic tissues as integrated functional units. The reference frame shifts with the focal subsystem, consistent with scale relativity in systems biology.

**Figure S2 Plant cell wall sensing and intercellular transport regulatory network.** (A) Cell wall components (cellulose, RG-II, RG-I, HG, extensin, etc.) interact with plasma membrane receptors (MCA1, LRR, EGF-like, COB, Malectin, receptor-like kinases, etc.), triggering signaling modules including RLCKs, CPKs, MAPKs, and GTPases to regulate cellular responses at transcriptional and post-translational levels. (B) Plant intercellular transport pathways and specialized structure regulation, covering apoplastic pathway, symplastic pathway (plasmodesmata), and molecular mechanisms underlying callose deposition (restricting plasmodesmata pore size), calcium accumulation (mediating intercellular signaling), and Casparian strip formation (controlling root radial transport). Abbreviations: COB, COBRA; CPKs, calcium-dependent protein kinases; EGF-like, epidermal growth factor-like; ER, endoplasmic reticulum; flg22, flagellin 22; GTPases, guanosine triphosphatases; HG, homogalacturonan; LRR, leucine-rich repeat; MAPKs, mitogen-activated protein kinases; MCA1, mid1-complementing activity 1; PD, plasmodesmata; PM, plasma membrane; RG-I, rhamnogalacturonan I; RG-II, rhamnogalacturonan II; RLCKs, receptor-like cytoplasmic kinases; ROS, reactive oxygen species; WT, wild type.
